# The effectiveness of case management for cancer patients: an umbrella review

**DOI:** 10.1186/s12913-022-08610-1

**Published:** 2022-10-14

**Authors:** Nina Wang, Jia Chen, Wenjun Chen, Zhengkun Shi, Huaping Yang, Peng Liu, Xiao Wei, Xiangling Dong, Chen Wang, Ling Mao, Xianhong Li

**Affiliations:** 1 Department of Respiratory Medicine, Xiangya Hospital, Central South University, Changsha, China; 2 National Clinical Research Center for Geriatric Disorders, Xiangya Hospital, Changsha, China; 3 Xiangya School of Nursing, Central South University, Changsha, China; 4 School of Nursing, University of Ottawa, Ottawa, Canada; 5 Center for Research on Health and Nursing, University of Ottawa, Ottawa, Canada; 6 Intensive Care Unit of Cardiovascular Surgery Department, Xiangya Hospital, Central South University, Changsha, China; 7 The 956th Army Hospital, Linzhi, China; 8 School of Nursing, Changsha Medical University, Changsha, China

**Keywords:** Case management, Cancer patients, Umbrella review, Health care, Outcome assessment, Quality of life

## Abstract

**Background:**

Case management (CM) is widely utilized to improve health outcomes of cancer patients, enhance their experience of health care, and reduce the cost of care. While numbers of systematic reviews are available on the effectiveness of CM for cancer patients, they often arrive at discordant conclusions that may confuse or mislead the future case management development for cancer patients and relevant policy making. We aimed to summarize the existing systematic reviews on the effectiveness of CM in health-related outcomes and health care utilization outcomes for cancer patient care, and highlight the consistent and contradictory findings.

**Methods:**

An umbrella review was conducted followed the Joanna Briggs Institute (JBI) Umbrella Review methodology. We searched MEDLINE (Ovid), EMBASE (Ovid), PsycINFO, CINAHL, and Scopus for reviews published up to July 8th, 2022. Quality of each review was appraised with the JBI Critical Appraisal Checklist for Systematic Reviews and Research Syntheses. A narrative synthesis was performed, the corrected covered area was calculated as a measure of overlap for the primary studies in each review. The results were reported followed the Preferred reporting items for overviews of systematic reviews checklist.

**Results:**

Eight systematic reviews were included. Average quality of the reviews was high. Overall, primary studies had a slight overlap across the eight reviews (corrected covered area = 4.5%). No universal tools were used to measure the effect of CM on each outcome. Summarized results revealed that CM were more likely to improve symptom management, cognitive function, hospital (re)admission, treatment received compliance, and provision of timely treatment for cancer patients. Overall equivocal effect was reported on cancer patients’ quality of life, self-efficacy, survivor status, and satisfaction. Rare significant effect was reported on cost and length of stay.

**Conclusions:**

CM showed mixed effects in cancer patient care. Future research should use standard guidelines to clearly describe details of CM intervention and its implementation. More primary studies are needed using high-quality well-powered designs to provide solid evidence on the effectiveness of CM. Case managers should consider applying validated and reliable tools to evaluate effect of CM in multifaced outcomes of cancer patient care.

**Supplementary Information:**

The online version contains supplementary material available at 10.1186/s12913-022-08610-1.

## Background

Cancer ranks as one of the leading causes of premature death among population around 30–69 years old across 134 countries [1], and the global incidence of cancer is about to reach 30.2 million new cases and 25.7 million deaths by 2040 [2]. Earlier detection and diagnosis, and development of diverse cancer treatments have increased the survival rate of cancer patients. According to Quaresma et al. [3], the cancer survival in the UK has doubled over the last 40 years alongside the advancement in cancer diagnosis and treatment. However, number of challenges exist in the current cancer care all over the world. Many cancer patients oftentimes receive a series of long-running and exhausting multi-modal treatments and experience descent in psychological, physical and social functioning, which have a significant negative impact on their quality of life (QoL) [4, 5]. In addition, the significant healthcare spending and productivity losses of cancer patients lead to a heavy patient economic burden, which is another substantial issue with cancer care [6]. A systematic approach is needed to mobilize and deliver appropriate resources, provide accessible, safe, and well-coordinated care for cancer patients received stressful treatments and shouldered heavy economic burden [7].

Case management (CM) is defined by the Case Management Society of America (CMSA) as “a collaborative process of assessment, planning, facilitation, care coordination, evaluation, and advocacy for options and services to meet an individual’s and family’s comprehensive health needs through communication and available resources to promote quality, cost-effective outcomes” (P. 11) [8]. According to the definition, CM is designed to use resources effectively to improve the quality of treatments, patient care services, and QoL of patients while reducing the relevant healthcare costs.

With the worldwide utilization of CM in cancer patient care, studies examining the effect of CM in improving patient-related outcomes or healthcare service use outcomes have been skyrocketing. Numbers of systematic reviews and meta-analyses have been published to synthesis the effectiveness of CM in recent years and often arrive at discordant conclusions. For example, Joo et al. [9] retrieved and synthesised results from nine experimental studies and found that CM effectively improved patients’ QoL and symptom management. While Aubin et al. [10] reported equivocal effect on both QoL and symptom management. Chan et al. [11] reported that four of the five randomized controlled trials showed insignificant impact of CM on patients’ QoL. The inconsistent evidence on the impact of CM may confuse or mislead the future case management development and relevant policy making. Considering the exist of several systematic reviews and research synthesis available to inform the application of case management for cancer patient care improvement, umbrella review could now be undertaken to compare and contrast published reviews and to highlight the consistent or contradictory findings around the effect of CM on manifold aspects of cancer patient care [12]. Thus, the current review was conducted to 1) synthesis systematic reviews that assess the effects of CM on cancer patient outcomes (e.g., QoL, functioning status, symptom management, satisfaction, etc.) and health care utilization outcomes (e.g., cost, hospital admissions, length of stay, treatment received compliance, etc.), 2) summarize measurement used in evaluating patient outcomes and health care utilization outcomes.

## Methods

### Design

This umbrella review followed the Joanna Briggs Institute (JBI) Umbrella Review (UR) methodology [12] and adhered to the Preferred Reporting Items for Overviews of systematic reviews (PRIO) checklist (see Additional file 1) [13]. This review has been registered with the Open Science Framework (10.17605/OSF.IO/7YQAP).

### Study searching methods

We performed literature search in five databases including MEDLINE (Ovid), EMBASE (Ovid), PsycINFO, CINAHL, and Scopus from inception to July 2022. Ethical approval and patient consent were not necessary since all analyses were based on previously published articles. The searching strategies in all five databases were developed with the help of a health science librarian. See Additional file 2 for the searching strategy and results in MEDLINE (Ovid). The studies were selected using the following inclusion and exclusion criteria.

### Inclusion and exclusion criteria

#### Population

Individuals diagnosed with any type of cancer at any cancer stages (early to advanced). Reviews targeted on people with no specified cancer diagnose were excluded.

#### Intervention

Case management interventions targeted on cancer patients. Case management is defined as a “collaborative process of assessment, planning, facilitation, care coordination, evaluation, and advocacy for options and services to meet an individual’s and family’s comprehensive health needs through communication and available resources to promote quality, cost-effective outcomes” [8]. Only reviews in which the effectiveness of CM as defined above was analyzed separately from other interventions were considered.

#### Comparison

Individuals in comparison groups received “treatment as usual” (TAU). TAU may include various interventions called “standard of care,” “usual care,” or “standard treatment,” but generally refers to treatment as it is commonly provided. Only studies that compared case management with “TAU” were selected.

#### Outcomes

Patient outcomes (e.g., quality of life, symptom management, functioning status), health care utilization outcomes (e.g., cost, hospital admissions, length of stay), etc.

#### Setting

Acute care hospitals and primary care settings (e.g., long-term care, nursing homes, community care services). Hospital was defined as any department of internal medicine or surgery as well as unspecified hospital settings.

#### Study design

Systematic review/meta-analysis that only included quantitative studies. We excluded studies full-texts unavailable online.

### Study selection

All retrieved studies were imported into Covidence systematic review software [14] and the duplicates were removed. Then, titles and abstracts were independently assessed by two researchers (XW and XD) according to the inclusion criteria. After that, the full texts of the selected abstracts were obtained and reviewed by the same two researchers (XW and XD) independently. The reference list of included studies was reviewed and searched for additional studies. Any disagreement between the two researchers were resolved through consultation with a senior researcher (PL).

### Quality appraisal for included reviews

Two reviewers (NW and LM) independently assessed the methodological quality of the individual studies using the JBI Critical Appraisal Checklist for Systematic Reviews and Research Syntheses [15]. The tool aims to determine the extent to which the review has addressed the possibility of bias in its design, conduct and analysis [15]. It consists of 11 criteria scored as yes, no, unclear, or not applicable. We adopted a scoring system used in previously published systematic reviews [16, 17]. For each article, a rating score was derived by taking the number obtained in the quality rating and dividing it by the total number of possible points allowed, giving each manuscript a total quality rating between 0 and 1. Studies were then classified as low (0–0.25), low-moderate (0.26–0.50), moderate (0.51–0.75), or high (0.76–1.0).

### Data extraction

We developed the data extraction form based on the research questions, and extracted following information: characteristics of included reviews such as publication year range, whether conducted meta-analysis or not, type of cancer patients, age of population, type and number of primary studies included; intervention names, components, and duration; outcomes and evaluation tools used; author’s conclusions and interpretations. Two researchers (NW and LM) extracted data independently from all included articles into an Excel spreadsheet and another researcher (XL) verified it for accuracy.

### Data synthesis

We were unable to statistically pool outcomes due to the heterogeneity of outcomes of the included reviews. Therefore, we conducted a narrative synthesis [18] of the numerical data of individual studies outcomes. The studies were summarized and synthesised by two reviewers (NW and ZS) independently and double checked by a third author (HY). Following the JBI UR methodology [12], we used a summary table to present clear, specific, and structured results from the selected reviews, and then synthesised these results to identify broad conclusions. To summarized information about the interventions we coded data into features, components and delivery strategies, and inductively developed themes within each domain as they emerged from the studies. As suggested by Li and colleagues [19], we grouped outcomes into: global QoL of patients, functional status (i.e. physical, cognitive, emotional, role, social), symptom management, cost, hospital (re)admission, length of stay, treatment received compliance, provision of timely treatment.

For clarity the term ‘primary studies’ refers to the articles found within the included reviews. As several primary studies are included in more than one review, the overall results and conclusions of an overview can be biased. To assess this bias, the degree of overlap between reviews was calculated with the Corrected Covered Area (CCA) method. The details of the CCA calculation have been described by Pieper and colleagues [20] elsewhere. A CCA score of less than 5% is regarded as a slight overlap, 5–9.9% as moderate overlap, 10–14.9% as high overlap and over 15% as a very high level of overlap. This measure has been validated in which the number of overlapped primary publications has a strong correlation with the CCA [21].

## Results

### Search outcome

**Fig. 1:**
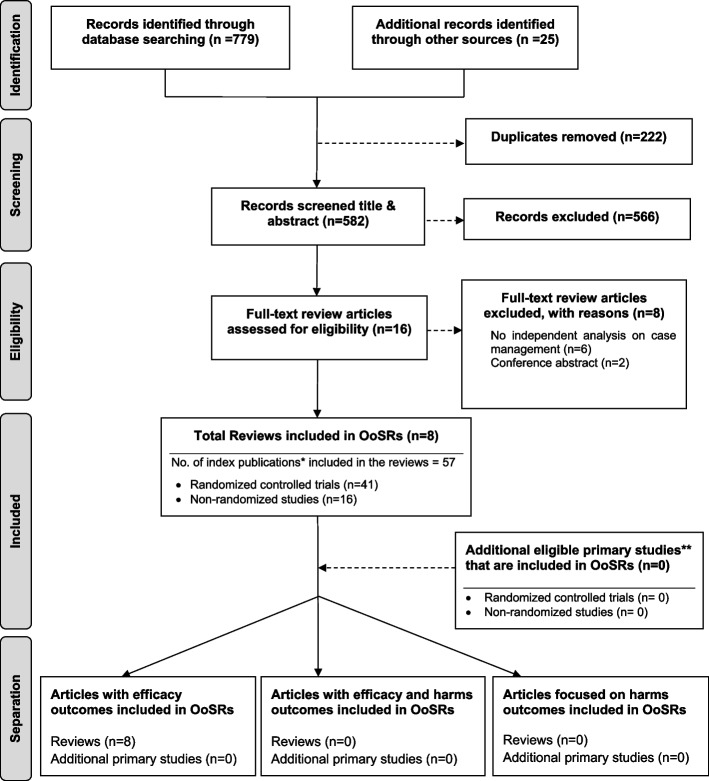
Flow chart for umbrella review. *Index publication is the first occurrence of a primary publication in the included reviews. **Additional eligible primary studies that had not been initially indentified by the search of the relevant reviews or obtained by updating the search of the included reviews

As shown in Fig. 1, our search strategy generated 804 potentially relevant records. Upon removing the duplicates, 582 studies screened by title and abstract, 16 were identified for full text screening. We excluded eight of the 16 studies for the following reasons: no independent analysis on the effect of case management (*n* = 6), or conference abstract (*n* = 2). The eight remaining systematic reviews were selected and assessed for methodological quality. In total, all the eight reviews included 57 primary studies, among which 12 were duplicated included in two or three reviews. Forty-one of the 57 primary studies were randomized controlled trials (see Additional file 3 for included primary studies).

### Methodological quality assessment

The quality assessment scores are presented in Table 1. Only one review was rated as moderate because not clarify whether two or more reviewers independently assessed the quality of included primary studies, and did not report the methods to minimize errors in data extraction or publication bias. The other seven reviews were rated as high quality. Despite rated as strong, the seven reviews still companied with one or two issues on the assessment of heterogeneity, search strategy, and recommendations for policy and/or practice.

**Table 1:** Methodological quality of included systematic reviews and studies

Author, year	1^a^	2	3	4	5	6	7	8	9	10	11	Quality rating^c^ (Total score)
Joo, 2019 [9, 22]	Y^b^	Y	Y	Y	Y	Y	Y	N	Y	Y	Y	Strong (0.91)
Wulff, 2008 [23]	Y	Y	Y	Y	Y	N	N	Y	N	Y	Y	Moderate (0.72)
Yin 2020 [24]	Y	Y	Y	Y	Y	Y	Y	Y	Y	Y	Y	Strong (1.00)
Li, 2014 [19]	Y	Y	Y	Y	Y	N	N	Y	Y	Y	Y	Strong (0.82)
Aubin, 2012 [10]	Y	Y	Y	Y	Y	Y	Y	Y	Y	Y	Y	Strong (1.00)
Chan, 2020 [11]	Y	Y	Y	Y	Y	Y	Y	Y	Y	N	Y	Strong (0.91)
Wu, 2021 [25]	Y	Y	Y	Y	Y	Y	Y	Y	Y	U	Y	Strong (0.95)
McQueen, 2017 [26]	Y	Y	Y	Y	Y	Y	Y	U	N	N	Y	Strong (0.77)

1. Is the review question clearly and explicitly stated?

2. Were the inclusion criteria appropriate for the review question?

3. Was the search strategy appropriate?

4. Were the sources and resources used to search for studies adequate?

5. Were the criteria for appraising studies appropriate?

6. Was critical appraisal conducted by two or more reviewers independently?

7. Were there methods to minimize errors in data extraction?

8. Were the methods used to combine studies appropriate?

9. Was the likelihood of publication bias assessed?

10. Were recommendations for policy and/or practice supported by the reported data?

11. Were the specific directives for new research appropriate?

^a^We applied the following 11 questions of JBI Critical Appraisal Checklist [1] for quality appraisal of the included reviews

^b^Y=Yes; N=No; U = Unclear

^c^Quality of reviews was classified as low (0–0.25), low-moderate (0.26–0.50), moderate (0.51–0.75), or high (0.76–1.0)

### Characteristics of included studies

Table 2 presents a descriptive summary of characteristics of the eight systematic reviews [9–11, 19, 23–26]. The eight reviews aimed to identify evidence of the effectiveness of CM on cancer patients. Three of the studies were a systematic review with meta-analysis [10, 25, 26]. Five of the eight reviews adhered to the PRISMA statement [11, 19, 24–26], two adopted Cochrane systematic review methodology [9, 10].

**Table 2:** Characteristics of the included reviews

• Review (First author, year) • Year range of original studies • Countries/areas	• Study designs in reviews • Methods • Meta- analysis	• Type of cancer patients • Total No. & Ranges of sample size • Mean age, Age range	• CM interventions • Components • Duration of interventions • Control group	• Note
**•** Joo, 2019 [9, 22] **•** 2008–2017 **•** Germany, Taiwan, UK, Turkey, Switzerland	**•** 3 RCTs, 6 controlled before-and-after study **•** Systematic Review adherence to Cochrane methods **•** No	**•** Breast (*n* = 2), colorectal (*n* = 1) and several types of cancer(*n* = 6) **•** 9601 & 18 to 7445 **•** 55.14, from 50.2–66.25 years	**•** Nurses led multidisciplinary case management **•** Palliative care, supportive services, and regular follow-ups **•** 4 weeks − 12 months **•** Usual care	**•** Diagnosed with cancer for at least 1 year and were in the middle of cancer therapy
**•** Wulff, 2008 [23] **•** 1989–2006 **•** USA & UK	**•** 7 RCTs **•** Systematic Review (not mentioned) **•** No	**•** Breast (*n* = 2), lung(*n* = 2) and different cancer types (*n* = 3) **•** 823 &166 to 379 **•** 66.31, from 45 to 92 years	**•** CM-like intervention **•** Multidisciplinary collaboration, care co-ordination, and in-person meetings between patient and the case manager aimed at supporting, informing and educating the patient. **•** 1–12 months **•** Standard medical care	**•** 15 cancers of the prostate or breast with metastasis
**•** Yin, 2020 [24] **•** 2000–2017 **•** Denmark, USA, Switzerland, UK, Turkey	**•** 7 RCTs **•** Systematic Review following PRISMA **•** No	**•** Breast(*n* = 2), colorectal (*n* = 1), and different kinds of cancer (*n* = 4) **•** 925 &18 to 280 **•** 56.10, from 27 to 87 years	**•** CM-like intervention **•** Coordination or multidisciplinary collaboration, in-person meeting or telephone contacting with patients, provide long term supports, education and information to the patients **•** 5–12 months **•** Routine care	**•** Especially advanced-stage cancer patients
**•** Li, 2014 [19] **•** 2003–2012 **•** USA, Canada, Denmark, UK, Taiwan	**•** 13 RCTs **•** Systematic Review following PRISMA **•** No	**•** Breast (*n* = 5), ovarian (*n* = 2), colorectal (n = 3), gastric cancer or hepatocellular carcinoma (*n* = 1), lung (n = 3) and multiple types of cancer (n = 2). **•** 3438 & 61 to 507 **•** 56.83, from 18 to 93 years	**•** CM focusing on single-skill training or symptom management **•** Single-skill training or symptom management, including hypnosis, foot reflexotherapy, cognitive behavioural stress management (CBSM) and energy and sleep enhancement; and multiple dimensional HRQOL or holistic case management **•** 4 days-24 months **•** Usual care	**•** /
**•** Aubin, 2012 [10] **•** 1981–2009 **•** UK, USA, Sweden, Taiwan, Australia, Canada	**•** 20 RCTs **•** Systematic review adherence to Cochrane methods **•** Yes	**•** Breast (*n* = 7), lung (*n* = 2), cervical/ ovarian (*n* = 1), prostate (n = 1), and all types of cancer(n = 7) **•** 4518 & 29 to 554. **•** 60.22, from 7 to 97 years	**•** Case management model of care **•** Mainly use strategies consisting in staff organization, arrangement for follow-up, and coordination of assessment and treatment. **•** 5 days-5 years **•** Usual care	**•** /
**•** Chan, 2020 [11] **•** 2011–2019 **•** USA, Norway, Hong Kong, South Korea, Denmark	**•** 5 RCTs **•** Systematic Review following PRISMA **•** No	**•** Breast cancer **•** 606 & 45 to 210 **•** 50.55, from 26 to 69	**•** Nurse-led case management **•** Requiring patient- initiated contact and interventions delivered face-to-face, by telephone and/or online by an experienced oncology nurse **•** 1–24 month **•** Usual care	**•** /
**•** McQueen, 2017 [26] **•** 1983–2013 **•** Netherlands, Scotland, England	**•** 2 RCTs, 1 controlled trial **•** Systematic Review following PRISMA **•** Yes	**•** Breast cancer (n = 2) & different kinds of cancer(*n* = 1) **•** 327 & 22 to 72 **•** 48.63, from 18 to 65 years	**•** Vocational case management approach **•** A wide range of assessments and support interventions, including counselling, functional capacity evaluation, work capability assessments, job analysis, and workplace adjustment such as modified work hours, modified work tasks, modified work environment and interventions designed to improve communication with managers. **•** 12–24 month **•** Usual care	**•** Working age adults
**•** Wu, 2021 [25] **•** 2003–2018 **•** US, Taiwan, Malaysia	**•** 1 RCT,1 quasi-experimental, 9 cohort **•** Systematic Review following PRISMA **•** Yes	**•** Breast (*n* = 8), gynecologic (*n* = 1) and a variety cancer (*n* = 2) **•** 7212 & 110 to 2252 **•** 51.51, from 16 to 89	**•** Nurse-led case management **•** Assessment, education, coordination, follow-up, consultation, support, and care team collaboration **•** Not reported **•** Usual care	**•** /

The eight reviews were published between 2008 and 2021, the primary studies in the reviews were published between 1983 and 2018. The number of primary studies regarding to CM included in each review ranged from three to 20. Five of the eight reviews included only randomized controlled trials (RCTs), the remaining reviews included a combination of study designs that involved RCTs, quasi-experimental and non-experimental studies (e.g., cohort study). The age of review participants ranged from 7 to 97 years and mean ages range from 48.63 to 66.31 years, which covers populations from children to elders. The total number of participants in each review ranged from 327 to 9601. Seven of the eight reviews included primary studies targeted on multiple types of cancer including breast, lung, colorectal, cervical, ovarian, prostate, gastric, hepatocellular, etc. Most of the primary studies included in the eight reviews were conducted in the United States, and there were also studies conducted in Canada, Australia, Europe (i.e., Germany, UK, Turkey, Switzerland, Denmark, Switzerland, Sweden, Norway, Netherlands) and East Asia (i.e., Hong Kong, Taiwan, South Korea, and Malaysia).

### CM interventions

**Fig. 2:**
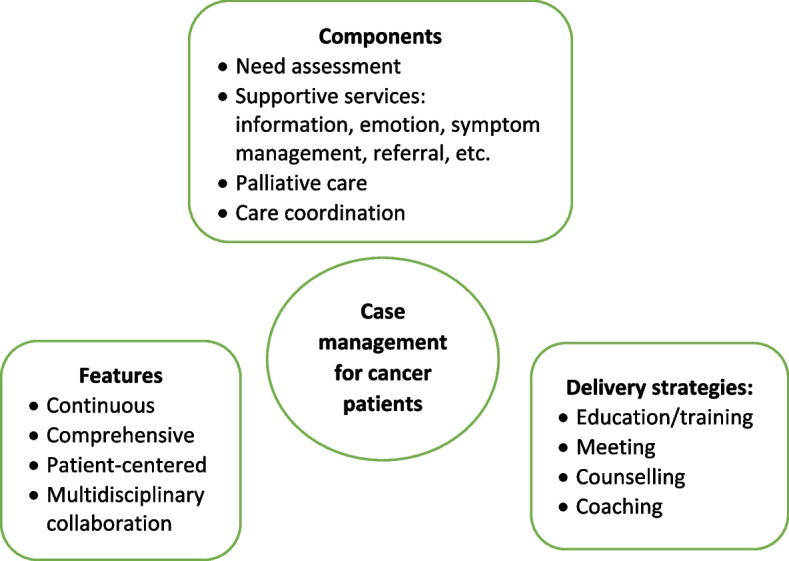
Features, components, and delivery strategies of case management for cancer patient care

As shown in Table 2, three studies reviewed trials of nurse-led CM interventions [9, 25, 26], two reviewed CM-like interventions that not termed as ‘CM’ while meet the CM definition by the CMSA [8, 23, 24]. Only one study reviewed CM focus solely on skill-training or symptom management [19]. All studies reviewed trials that facilitated the CM in a multidisciplinary collaboration approach. The duration of CM ranged from 4 days to 5 years. We presented the feature, components and delivery strategies of CM interventions for cancer patients in Fig. 2 by summarizing descriptions in each review. Congruent with the components defined by CMSA [8], all CM interventions included patient assessment, supportive services such as information and emotion support, care coordination by conducting education, consultation, and in-person, telephone or online coaching for regular follow-up. One critical component of CM interventions for cancer patients is the provision of palliative care. Control groups (CGs) of all studies reviewed in the reviews received usual treatment of care.

### Corrected Covered Area (CCA)

Table 3 presents the CCA for each outcome and as a whole. Overall, primary studies had a slight overlap across the eight reviews (CCA = 4.5%). In addition, no overlapping of primary studies was found for six of the 16 outcomes, including self-efficacy, psychological function, hospital (re)admissions, length of stay, and provision of timely treatment. Only one outcome (i.e., symptom management) showed slight overlap (0.7%). The CCA for other five outcomes (i.e., global QoL, physical function, role function, patient satisfaction, cost) evaluated by more than 2 reviews were between 5 to 9.9%, indicated a moderate overlap. The CCA for survivor status, cognitive function, emotional function, and treatment received compliance were over 10%.

**Table 3:** Corrected Covered Area (CCA) for outcomes

Outcomes	No. of reviews reported the outcome (c)	Sum of the number of primary studies (N)	Total number of primary studies (r)	CCA^a^ (%)
Global Quality of Life	7	39	28	6.5
Functional status				
• Psychological function	4	18	18	0
• Physical function	3	7	6	8.3
• Role function	4	6	5	6.7
• Cognitive function	5	9	5	20
• Emotional function	3	7	5	20
• Social function	1	5	5	/
Symptom management	7	24	23	0.7
Self-efficacy	2	4	4	0
Survivor status	2	6	4	50
Patient satisfaction	5	11	9	5.6
Cost	5	11	8	9.4
Hospital (re)admissions	2	4	4	0
Length of stay	3	5	5	0
Treatment received compliance	4	7	5	13.3%
Provision of timely treatment	1	5	5	/
Overall	8	75	57	4.5%

N = the sum of the number of primary studies in each review

r = the total number of primary studies

c = the number of reviews

less than 5%, slight overlap; 5–9.9%, moderate overlap; 10–14.9%, high overlap; over 15%, very high level of overlap [2]

^a^$$\textrm{Corrected}\ \textrm{Covered}\ \textrm{Area}\ \left(\textrm{CCA}\right)=\frac{N-r}{rc-r}$$

### Measurement used

Table 4 presents the quantitative measurement used in primary studies. As shown in Table 4, studies investigated global QoL using different QoL-related scales, among which Functional Assessment of Cancer Therapy (FACT) (used in 15 primary studies) were most frequently applied, followed by the European Organisation for Research and Treatment of Cancer Core Quality of Life Questionnaire 30 (EORTC QLQ-C30) (used in 11 primary studies), and short form health survey (i.e., SF-8, SF-12, SF-36) (used in 10 primary studies). Different types of FACT tool were used according to the cancer types. For example, FACT-G was used for general cancer patients assessment, and FACT-B was used to evaluate breast cancer-related QoL. For the assessment of overall symptom management, SF-36 and Symptom Distress Scale (SDS) were used most frequently (used in four primary studies each). Different dimensions of SF-36 were also applied to evaluate other outcomes such as physical, emotional, and social function. Hospital Anxiety and Depression Scale (HADS) was the top employed tool in measuring the psychological function of patients. Patients’ sick leave days and the number of patients return to work were top employed metrics to evaluate the role function of patients. No unified tools were utilized to assess patient satisfaction towards the CM and majority of the primary studies used self-developed questionnaires.

**Table 4:** Measurements used in primary studies

Outcomes		Measurements (No. of primary studies used the measurement)
Global Quality of Life		FACT-G (*n* = 8) FACT-E (*n* = 1) FACT-B (*n* = 5) FACT-L (*n* = 1) EORTC QLQ-C30 (*n* = 11) SF-36 (*n* = 5) SF-12(*n* = 4) SF-8 (*n* = 1) EORTC-11 (*n* = 2) Spitzer Quality of Life Index (*n* = 2) SDS (*n* = 2) PHQ (*n* = 1) ESDS (*n* = 1) HADS (*n* = 1) MUIS (*n* = 1) Karnofsky Performance Status (*n* = 1) Visual analogue scale (*n* = 1)
Functional status (i.e. Physical, Cognitive, emotional, role, social)	Psychological function	HADS (*n* = 8) CES-D: depression (*n* = 4) Impact of Event Scale (*n* = 2) SF-36 (*n* = 1) CARES-SF (*n* = 1) Brief Symptom Inventory: anxiety (*n* = 1) Depressive symptom subscale of the POMS (*n* = 1) Hamilton rating scale for anxiety (*n* = 1) Affects Balance Scale (*n* = 1) Cancer Rehabilitation Evaluation (*n* = 1) 28-item general health questionnaire (*n* = 1)
	Physical function	SF-36 (*n* = 2) Objective assessment of arm functions (*n* = 1) IPAQ leisure-time activity subscale and records (*n* = 1)
	Role function	Sick leave days (*n* = 4) Rate/numbers of return to work(*n* = 4) Social adjustment (*n* = 1) Employment patterns (*n* = 1) IPAQ leisure-time activity subscale (*n* = 1)
	Cognitive function	MUIS (*n* = 5) IPAQ leisure-time activity subscale (*n* = 1)
	Emotional function	POMS (*n* = 4) SF-36 five-item mood state score & role-emotional and mental health subscales (*n* = 2) EORTC C-30 (*n* = 1)
	Social function	SF-36 (*n* = 1) Social Support Questionnaire (*n* = 1) The Dyadic Satisfaction and Dyadic Cohesion subscales from the 32-item Spanier Dyadic Adjustment Scale (*n* = 1)
Symptom management	Symptom overall	SF-36 (*n* = 4) SDS (*n* = 4) Profile of Mood States questionnaire (*n* = 2) SCL-20 (*n* = 1) SCL-90 (*n* = 1) ESDS (*n* = 1) PHQ (*n* = 1) Standard questions on symptom severity (*n* = 1) Inventory of Current Concerns (*n* = 1) Physiologic complication classification (*n* = 1) Memorial Symptom Assessment Scale (*n* = 1) Edmonton Symptom Assessment System (*n* = 1) CES-D (*n* = 1) Chemotherapy Symptom Assessment Scale (*n* = 1) Distress thermometer (*n* = 1)
	Pain	McGill pain questionnaire (*n* = 2) Visual analogue scale (*n* = 2) Intensity subscale of the Brief Pain Inventory (*n* = 1)
	Fatigue	FACIT-F (*n* = 2) Brief Fatigue Inventory (*n* = 2) General Fatigue Scale (*n* = 1) Fatigue symptom subscale (*n* = 1)
	Sleep	Pittsburgh Sleep Quality Index /Inventory: Sleep (*n* = 1)
Self-efficacy		General Self-Efficacy Scale (*n* = 1) Cancer Behavior Inventory (*n* = 1) Strategies Used by Patients to Promote Health (*n* = 1) Self-developed questionnaire (*n* = 1)
Survivor status (e.g., Length of survival)		Medical records (*n* = 5)
Patient satisfaction		Self-developed patient satisfaction questionnaire (*n* = 7) Patient satisfaction (*n* = 1) Medical Outcomes Study-Patient Satisfaction Questionnaire (*n* = 1) Satisfaction and Accessibility scales (*n* = 1)
Cost		Billing systems (*n* = 4) Health care costs (*n* = 3) Medical records (*n* = 3) Time logs (*n* = 1) Euro Qol 5D (*n* = 1)
Hospital (re)admissions		Patients’ number of hospital admissions (*n* = 4) ICU admission rate (*n* = 1)
Length of Stay /hospitalizations		length of stay in hospital/ ICU (*n* = 2) Medical records audit (*n* = 2) Hospitalization: Any episode of client hospitalization which respired an overnight stay (*n* = 1) Referral rate: Number of cancer patients referred to home care per 100,000 population (*n* = 1)
Treatment received compliance (e.g., intention, acceptance, completion)		Therapy acceptance rate (*n* = 6) Therapy completion rate (*n* = 3) Medical records audit (*n* = 1) Rate of patient continuing treatment (*n* = 1) Patient Assessment of Chronic Illness Care (*n* = 1)
Provision of timely treatment		Time from diagnosis to treatment (*n* = 3)

*CES-D* Center for Epidemiological Studies-Depression Scale, *EORTC-11* European Organization for Research and Treatment of Cancer 11, *EORTC QLQ-C30* European Organisation for Research and Treatment of Cancer Core Quality of Life Questionnaire 30, *ESDS* Enforced Social Dependency Scale, *FACT-B* Functional Assessment of Cancer Therapy - Breast Cancer, *FACT-E* Functional Assessment of Cancer Therapy- Esophagus, *FACT-G* Functional Assessment of Cancer Therapy- General, *FACT-L* Functional Assessment of Cancer Therapy Scale-Lung, *FACIT-Fatigue* Functional Assessment of Chronic Illness Therapy Fatigue Scale, *HADS* Hospital Anxiety and Depression Scale, *IPAQ* International Physical Activity Questionnaire, *KPS* Karnofsky Performance Status, *MUIS* Mishel Uncertainty in Illness Scale, *PHQ* Personal Health Questionnaire, *SCL-20* Symptom Checklist-20, *SCL-90* Symptom Checklist-90, *SDS* Symptom Distress Scale, *SF-8* Medical Outcomes Study 8-item short form health survey, *SF12* Medical Outcomes Study 12-item short form health survey, *SF-36* Medical Outcomes Study 36-item short form health survey

### Effect of CM on patient and health care utilization outcomes

The main outcomes from the seven systematic reviews are presented and summarized in Table 5. Seven of the eight reviews reported the effects of case management on patients’ global QoL and showed mixed findings. Around half (49%, 19/39) of the primary studies included in the seven reviews reported significant positive impact of CM on global QoL. As for the functional status, there was a strong concordance among primary studies regarding the effectiveness of CM in improving cognitive function (e.g., uncertainty, health perceptions) (89%, 8/9); Equivocal effects were reported on psychological (e.g., patient anxiety, depression), physical (e.g., arm function), role function (e.g., sick leave days, patients returning to work), emotional (e.g., mood) and social function (e.g., social support) [9, 11, 26]. The findings regard to symptom management were more positive, with 75% (18/24) primary studies included in seven reviews revealed significant positive impact of CM on symptom severity and symptom distress decrease of pain, nausea, fatigue, discomfort, etc. Three of the four primary studies in two reviews [9, 11] showed no significant influence of CM on patients’ self-efficacy. Wulff et al. [23] and Aubin et al. [10] reported mixed findings on the impact of CM on survivor status, with four of the six primary studies reported significant positive impact. The effect of CM on patient satisfaction was reported in five reviews and showed mixed results.

**Table 5:** Effect of case management on patient and healthcare utilization outcomes

Outcomes		Author, year	Findings	No. (%) of primary studies reported positive results
Global Quality of Life		Wulff, 2008 [23]	2/3 RCTs reported some dimensions of QoL (e.g., well-being) among CM patients showed significant higher improvement than CG (*p* < .05).	19/39 (49%) positive
		Joo, 2019 [9, 22]	2/3 RCTs reported significant greater improvement of generic and cancer specific QoL among CM group than CG (*p* < .05). 1/2 quasi-experimental study reported significant better improvement of QoL in CM group than CG (*p* < .05).	
		Yin, 2020 [24]	5/7 RCTs reported significant improvement QoL of cancer patients in CM group (*p* < .01).	
		Li, 2014 [19]	4/8 RCTs reported improved general QoL (*p* < .05), social and functional well-being (*p* = .01), mental and physical QoL (*p* = 0.03) in CM group.	
		Chan, 2020 [11]	1/2 RCT reported equivocal effects on HRQoL during treatment. 2/2 RCTs reported no difference in HRQoL during survivorship. 1/1 RCT reported superior effects on disease specific HRQoL, but only for unmarried women at one month during diagnosis to survivorship (*p* < .05).	
		Aubin, 2012 [10]	4/9 RCTs showed significant improvement of QoL in CM group than CG (*p* < .05).	
		McQueen, 2017 [26]	2/2 RCTs showed no differences in QoL, though 1 RCT reported a trend of increased quality of life at six month follow up though at 12 months follow up.	
Functional status	Psychological function	Joo,2019 [9, 22]	1/1 quasi-experimental study reported no difference in anxiety and depression between CM and CG.	8/18 (44%) positive
		Li, 2014 [19]	4/6 RCTs reported significant effects on emotional upset, intrusive thoughts, anxiety, and depression in CM group (*p* < .05).	
		Chan, 2014 [11]	3/3 RCTs reported no significant difference in anxiety and depression between CM group and control group.	
		Aubin, 2012 [10]	3/6 RCT reported significant reduction in depression in CM group (*p* < .05). 1/1 RCT reported significant reduced psychological morbidity in CM group (*p* < .05). 1/1 RCT reported no significant differences of psychosocial functioning in CM group.	
	Physical function	Wulff, 2008 [23]	1/1 RCT reported significant improved arm function in CM group than CG (*p* = 0.037).	4/7 (57%) positive
		Aubin, 2012 [10]	1/1RCT reported significant long-term improvements in sexual functioning in CM group (*p* < .05). 1/1 RCT reported significantly higher percentage of normal arm function two months after surgery in CM group (*p* < .05). 1/1 RCT reported significant improvements in physical functioning (*p* < .05).	
		Chan, 2020 [11]	3/3 RCTs reported equivocal effects on physical activity compared with usual care during survivorship.	
	Role function	Joo, 2019 [9, 22]	1/1 RCT reported non-significant difference in sick leave days post-surgery between CM and control group (*p* = 0.122).	2/6 (33%) positive
		McQueen, 2017 [26]	2/2 RCTs reported CM have some positive impact on return to work rates, while meta-analysis showed no significant differences of patient numbers returning to work. 2/3 studies (2 RCTs, 1 controlled trial) reported a trend of fewer days in CM group, while no significant difference was found in sick leave days. 1/1 controlled trail reported less problems with social adjustment and returning to house work in CM group than CG. 1/1 RCT reported no discernible difference in the pattern of changes to working hours.	
		Aubin, 2012 [10]	1/1 RCT reported significant reduction in physical role impact (*p* < .05).	
		Chan, 2020 [11]	1/1 RCT reported no significance between groups in role function.	
	Cognitive function	Wulff, 2008 [23]	1/1 RCT reported uncertainty among CM patients showed significant higher change than CG (*p* < .05).	8/9 (89%) positive
		Li, 2014 [19]	1/1 RCT reported significant better improvement in uncertainty in CM group (*p* < .05).	
		Yin, 2020 [24]	2/2 RCTs reported significant decreased uncertainty in CM group (*p* < .05).	
		Chan, 2020 [11]	1/1 RCT reported no significance between groups in cognitive function.	
		Aubin, 2012 [10]	3/3 RCTs reported significant decrease in uncertainty (*p* < .05). 1/1 RCT reported significant differences in health perceptions (*p* < .05).	
	Emotional function	Wulff, 2008 [23]	1/3 RCTs reported significant higher improvement in mood among CM patients than CG (*p* < .01).	4/7 (57%) positive
		Aubin, 2012 [10]	2/3 RCTs reported significant better scores for emotional functioning in CM group (*p* < .05).	
		Chan, 2020 [11]	1/1 RCT reported greater improvements in mood disturbance in CM group at the first and third month during diagnosis to survivorship.	
	Social function	Aubin, 2012 [10]	1/1 RCT reported significant improved social functioning in CM group (*p* < .05). 1/3 RCT reported higher support by family and friends as well as a significant increase in the overall social support and nurse/physician social support in CM group (*p* < .05). 1/1 RCT reported dyadic adjustment did not differ statistically from the CG.	2/5 (40%) positive
Symptom management		Joo,2019 [9, 22]	1/1 quasi-experimental study reported significant more decrease in symptom severity in the CM group than CG (*p* < .000).	18/24 (75%) positive
			1/1 RCT reported no significant differences in self-reported levels of fatigue between CM and control group.	
		Wulff, 2008 [23]	2/3 RCTs reported significant less symptom distress, enforced social dependency in CM group (P = 0.03).	
		Li, 2014 [19]	3/4 RCTs reported significant less pain, nausea, fatigue, discomfort in CM group than CG (*p* < .05).	
		Chan, 2020 [11]	2/2 RCTs reported superior effects on symptom burden outcomes during treatment and survivorship in CM group.	
		McQueen, 2017 [26]	1/1 RCT reported no significant differences in self-reported levels of fatigue.	
		Aubin, 2012 [10]	3/6 RCTs reported significant differences in symptoms and symptom control between CM group and CG (*p* < .05). 2/4 RCTs reported significant improved pain control in CM group (*p* < .05). 1/1 RCT reported significantly less severe dyspnoea and peripheral neuropathy in CM group (*p* < .05). 1/1 RCT reported no significant difference in fatigue.	
Self-efficacy		Joo,2019 [9, 22]	1/1 RCT reported significant difference in self-efficacy between CM and control group (*p* < .01).	1/4 (25%) positive
		Chan, 2020 [11]	2/2 RCTs reported equivocal effects on self-management/behavioural outcomes during treatment. 1/1 RCT reported no differences in self-efficacy between groups.	
Survivor status (e.g., Length of survival)		Wulff, 2008 [23]	1/2 RCT reported significant higher 2-year survival rate of late-stage patients in CM group (*p* < .05), and significantly more patients in CM group died at home (*p* < .05).	4/6 (67%) positive
		Aubin, 2012 [10]	3/4 RCT reported significant increased survival in CM group (*p* < .05).	
Patient satisfaction		Joo, 2019 [9, 22]	1/1 quasi-experimental study reported significant higher satisfaction level of patients and family in the CM group (*p* < .05).	6/11 (55%) positive
		Wulff, 2008 [23]	3/3 RCTs reported significant higher patient satisfaction in intervention group (*p* < .05).	
		Li, 2014 [19]	1/1 RCT reported no significant higher patient satisfaction in CM group over control group.	
		Aubin, 2012 [10]	4/5 study RCTs reported no significant difference in patient satisfaction with care and service use.	
		Chan, 2020 [11]	1/1 RCT reported significant improvements in satisfaction with treatment and rehabilitation in CM group (*p* < .05).	
Cost		Joo, 2019 [9, 22]	1/1 quasi-experimental study reported no significant difference in direct health costs between CM group and control group. 1/2 controlled before-and-after study reported significant difference in monthly cancer-related medical costs between CM and control group (*p* < .05), 1/2 controlled before-and-after study reported no significant difference in total costs.	1/11 (10%) positive
		Wulff, 2008 [23]	2/2 RCTs reported no significant difference in program contact, salary, overall costs, etc., between CM group and control group.	
		Yin,2020 [24]	2/2 RCTs reported no significant difference in health care costs (e.g., reimbursements or overall charges).	
		Aubin, 2012 [10]	2/2 RCTs reported no significant difference in costs between CM and control groups.	
		Chan, 2020 [11]	1/1 RCT reported a significantly lower cost per person in the 6-cycle chemotherapy subgroup (*p* < .05) and no difference in health service utilization during treatment. 1/1 RCT reported no difference in overall cost during diagnosis to survivorship.	
Hospital (re)admissions		Joo, 2019 [9, 22]	1/1 quasi-experimental study reported unplanned readmission rate caused by infection significantly decreased in the CM group compared with the CG (1.5% vs. 4.7% in the CG, *p* = .002). 2/2 controlled before-and-after study reported CM group had significant lower inpatient and ICU admission rate than control group (*p* < .05).	3/4 (75%) positive
		Wulff, 2008 [23]	1/1 RCT reported no significant difference in hospital admission or readmission rates between CM and control group.	
Length of Stay (LOS) /hospitalizations		Joo, 2019 [9, 22]	1/1 quasi-experimental study reported no significant difference in length of stay between CM and control groups. 1/1 controlled before-and-after study reported no significant difference in ICU days between CM and control group.	1/5 (20%) positive
		Wulff, 2008 [23]	2/2 RCTs reported no significant change in length of stay	
		Aubin, 2012 [10]	1/1 RCT reported no significant change in hospitalization, while reported significant increase in cancer patient referrals to home care.	
Treatment received compliance (e.g., intention, acceptance, completion)		Wulff, 2008 [23]	1/1 RCT reported more cancer-specific therapies (e.g., breast-conserving surgery, radiation therapy) received in CM group than control group (*p* < .05).	7/7 (100%) positive
		Joo, 2019 [9, 22]	1/1 quasi-experimental study reported the rate of patient continuing treatment in the institution significantly increased in the CM group than the control group (93.8% vs. 84.8%, in the CG, *p* < .001). 1/1 RCT reported the accordance of care increased by 0.20 in the CM group and decreased by 0.29 points in the CG (*p* = .009).	
		Wu, 2021 [25]	3/3 studies (1 RCT & 2 cohort studies) reported a significant 60% higher hormone therapy acceptance rate, but no significant difference in chemotherapy and radiotherapy, with a combined acceptant rate of more than 61 and 142%.	
			Meta-analysis of 3 studies (1 quasi-experimental study and 2 cohort studies) showed a significantly higher treatment completion rate than control group.	
		Aubin, 2012 [10]	1/1 RCT reported that older women with breast cancer were significantly more likely to receive breast-conserving surgery, and those women who received breast-conserving surgery were more likely to receive adjuvant radiation therapy in CM group.	
Provision of timely treatment		Wu, 2021 [25]	5/5 cohort studies reported a decrease in the time from diagnosis to treatment (from 3.8 to 17.2 days) in intervention group, and had statistically significant shorter time than control group.	5/5 (100%) positive

*CM* case management, *CG* control group, *RCT* Randomized Controlled Trial

Of the eleven primary studies reported cost, only one controlled before-and-after study in Joo et al.’s [9] review reported significant impact on monthly cancer-related medical costs. The evidence concerning patients’ length of stay yielded no significant findings. Overall significant positive effect was reported on hospital (re)admission (e.g., inpatient and ICU admission rate), treatment received compliance (e.g., therapy acceptance or completion rate), and provision of timely treatment.

## Discussion

This umbrella review is the first to summarize the results of systematic reviews that synthesised the evidence on the effectiveness of CM on cancer patient outcomes and relevant health care utilization. Most reviews (7/8) showed a high methodological quality. Different tools were used to measure the effect of CM on the same outcome. The evidence regards to the effectiveness of CM is mixed. The summarized results revealed that CM was more likely to improve symptom management, cognitive function, hospital (re)admission, treatment received compliance, and provision of timely treatment for cancer patients. Overall equivocal effect was reported on cancer patients’ global QoL, psychological, physical, role, emotional and social function, self-efficacy, survivor status, and patient satisfaction.

No universal tools were used to measure improvement of each outcome in the CM group compared with the control group, making it challenging to conduct a meta-analysis of studies results [22, 27]. This is a common issue faced the included reviews. Five of the eight reviews failed to conduct meta-analysis due to the heterogeneity [9, 11, 19, 23, 24]. Joo and Huber [22] conducted a review of reviews on the effect of CM on health care utilization outcome of chronic illness patients, they recognized the same problem and suggested using valid and standardized tools to minimize the differences in measurements. Despite various tools used, our review showed that FACT, EORTC QLQ-C30, and short form health survey (i.e., SF 36, SF 12, and SF 8) were most frequently applied to measure the effect of CM on the global QoL of cancer patients. These tools were also used in evaluating specific dimensions of QoL such as psychological, physical, emotional, and social function. This aligned with previous reviews [28, 29] that found FACT and EORTC QLQ-C30 were the most common and well developed QoL instruments in cancer patients. FACT-G is considered appropriate for use with any types of cancer patients [30]. It is a 27-item tool that includes four primary QoL domains: physical well-being, social/family well-being, emotional well-being, and functional well-being [31]. Other versions of FACT (FACT-B [32], FACT-L [33] and FACT-E [34]) for specific type of cancer patients were developed by incorporating the four dimensions of FACT-G with additional cancer type-specific questions. EORTC QLQ-C30 was another type of QoL assessment tools for cancer patients specifically. It was developed by Aaronson et al. [35] and contains four domains: physical, emotional, cognitive and social functions, and a higher score indicates better QoL. The Short Form Health Survey is the most commonly used measure in evaluating QoL domains of patients suffering from a wide range of medical conditions [36]. Research found it provides reliable and valid indication of general health among cancer patients [37, 38].

QoL is the most frequently evaluated outcome in our review with 39 primary studies in seven reviews reported the global QoL of cancer patients. Joo et al. [9] found that CM interventions improved QoL of cancer patients. Yin and colleagues [24] revealed that cancer patients achieved better physical and psychological condition through symptom management, needs assessment, direct referrals, and other services in CM. However, summarized results in our review show that the CM had equivocal effect on cancer patients’ global QoL and dimensions including psychological, physical, role, emotional and social function. Cognitive function is the only dimension showed positive change. Despite CM interventions share similar definitions and principles [8]. It is hard to foresee which aspect(s) of CM interventions contribute to certain effects due to their comprehensiveness [24]. Yin et al. [24] argued that the control group may receive a higher quality treatment than planned usual care since all the participants were not blinded and they have been informed about the aim of the study. Indicating a more rigorous design and evaluation is needed to avoid this information bias.

In the meantime, included reviews claimed that few primary studies reported enough details about CM interventions, including model used [10, 11], dose and intensity [9, 19, 24], interventionist qualifications [11], protocol or manual used [9, 23], and fidelity [23]. Particularly, the COVID-19 pandemic has considerable influence on the care delivery for cancer patients. For example, the more frequently utilization of remote patient monitoring technologies that incorporate community resources, primary care and allied health disciplines, as well as clinics to keep cancer patients away from acute care hospitals as much as possible [39]. Many of these changes have been integrated within routine case management for cancer care during the pandemic [39]. It is well-needed to report how those CM intervention were conducted follow standard reporting guidelines, in order to provide recommendation for future research.

Our review showed that CM is likely to improve the symptom management. Eighteen of the 24 included primary studies reported positive effect of CM on symptom management, including decrease symptom distress or severity of fatigue, pain, nausea, and vomiting. The same positive effect on symptom management was also revealed in other types of patients. Joo and colleagues [40] found that CM reduced substance use and significantly influenced abstinence rates among populations experienced substance disorders. Reviews by Stokes et al. [27] and Welch et al. [41] revealed positive effect on symptom release among people with long-term conditions and diabetes patients, respectively. The multidisciplinary collaboration approach adopted [10], and availability of professional support post-hospitalization [9, 41] in CM might contribute to the improvement of symptom management. Specifically, multidisciplinary team involves physicians, nurses, and aligned healthcare professionals provides throughout and multifaced symptom assessment and management [10]. In addition, CM programs continuously follow up and advocate for patients’ concerns [8]. Specifically, case managers are available to patients 24 hours a day by phone call even after discharged, providing opportunity for immediate professional guidance on symptom management [9].

As for other patient outcomes, there is insufficient evidence of effect on self-efficacy and survivor status of cancer patients. Only three and four primary studies in total reported these two outcomes, respectively. Eleven primary studies in five reviews reported patient satisfaction and showed mixed results. Inconsistent results were found in a review of reviews by Buja et al. [7] which concluded strong evidence of CM improving satisfaction of patients with long term condition. In agreement with Joo and Huber’s [25] review, we found that CM favorably affect healthcare utilization outcomes such as treatment received compliance, hospital (re)admission, and provision of timely treatment. While the strength of the evidence was limited either by the high level of primary studies overlapping (CCA) (i.e., treatment received compliance, CCA = 13.3%) or the small number of studies reported certain outcomes (i.e., hospital admission, provision of timely treatment). Notably, the summarized results from included reviews conclude that despite theoretical benefits [8], in practice there is only slight evidence of benefits on reduction in the cost of care for cancer patients participated in CM interventions.

We provide some recommendations for future research based on the summarized results: 1) Future research should clearly describe details of CM intervention and its implementation, including theoretical underpinnings, dose and intensity, interventionist qualifications, protocol or manual used, fidelity, etc. In that way these details can be included in future systematic reviews, and effectiveness of individual elements of the intervention can be examined [27]. We recommend use standard guidelines to help organize the CM intervention reporting. For example, the Template for Intervention Description and Replication (TIDeiR) is one of the most popular guidelines that could be used to report the full breadth of CM interventions: from intervention rationale to assessments of treatment adherence and fidelity [42]. 2) More rigorous trials are needed to evaluate the effectiveness of CM. 3) Studies should also explore the barriers to and facilitators of CM implementation across various types of cancer patients at different stages, providing evidence for conducting successful CM implementation in the future.

## Strengths and limitations

We conducted an umbrella review instead of a meta-analysis due to the heterogeneity of review outcomes. Although an umbrella review can only show the tendency or direction of the effect of CM rather than providing the magnitude or significance level of influence [12], the current evidence on the effect of CM in cancer patients was comprehensively summarized. There were some challenges when conducting the review. First, the quality of the umbrella reviews was greatly affected by the quality of the original reviews [12]. In this study, we confirmed that the quality of the original reviews were mostly high as assessed by the JBI Critical Appraisal Checklist [15]. Second, if the primary studies were included in several reviews, they may produce bias related to overlapping effects [20]. By calculating the CCA, we showed that 75% (12/16) of the individual outcomes had no to moderate overlapping of primary studies between included reviews, revealing that these results from each review were relatively independent. Cautious are needed on the summarized evidence regards to the effect of CM on survivor status, cognitive function, emotional function, and treatment received compliance because of the high overlapping (CCA > 10) between the reviews reported those outcomes.

There are limitations in our review. The first limitation concerns that the searching was limited to English-language articles and did not access unpublished papers. Second, as suggested by the JBI UR methodology [12], we did not assess the quality of evidence from included reviews, it increased the uncertainty of the review findings.

## Conclusion

Effective CM aims to influence the health care delivery system in improving the health outcomes of cancer patients, enhancing their experience of health care, and reducing the cost of care. Our review found mixed effects of CM reported in cancer patient care. The summarized results revealed that CM was likely to improve symptom management for cancer patients. We also found CM has the tendency to enhance cancer patients’ experience of health care such as reducing hospital (re)admission rates, improving treatment received compliance and provision of timely treatment. Only slight evidence of benefits was reported on reducing the cost of care for cancer patients. Overall, more rigorous designed primary studies are needed to demonstrate the effects of CM on cancer patients and explore the elements of effective CM interventions.

## Supplementary Information

**Additional file 1.**

**Additional file 2.** Searching strategies.

**Additional file 3.**

## Data Availability

All data generated or analysed during this study are included in this published article and its supplementary information files.

## Abbreviations

CCA: Corrected Covered Area
CGs: Control groups
CM: Case management
CMSA: Case Management Society of America
EORTC QLQ-C30: European Organization for Research and Treatment of Cancer Core Quality of Life Questionnaire 30
FACT-B: Functional Assessment of Cancer Therapy - Breast Cancer
FACT-E: Functional Assessment of Cancer Therapy- Esophagus
FACT-G: Functional Assessment of Cancer Therapy- General
FACT-L: Functional Assessment of Cancer Therapy Scale-Lung
QoL: Quality of life
HADS: Hospital Anxiety and Depression Scale
JBI: Joanna Briggs Institute
PRISMA: Preferred Reporting Items for Systematic Reviews and Meta-Analyses
RCTs: Randomized controlled trials
SDS: Symptom Distress Scale
SF-8: Medical Outcomes Study 8-item short form health survey
SF12: Medical Outcomes Study 12-item short form health survey
SF-36: Medical Outcomes Study 36-item short form health survey
TAU: Treatment as usual
