# Supplementary material for: The effectiveness of case management for cancer patients: an umbrella review
Source: BMC Health Serv Res. 2022 Oct 14;22:1247. doi: 10.1186/s12913-022-08610-1 (PMC9562054; doi:10.1186/s12913-022-08610-1)
Supplement: Supplementary file 2 — Additional file 2. Searching strategies. [file 12913_2022_8610_MOESM2_ESM.docx]

**Additional file 2 Searching strategies**

Search strategies in Ovid MEDLINE:

Database: Ovid MEDLINE(R) ALL <1946 to July 08, 2022>

Search Strategy:

--------------------------------------------------------------------------------

| 1 | Neoplasms/ |
| --- | --- |
| 2 | Cancer*.ti,ab. |
| 3 | Neoplas*.ti,ab. |
| 4 | Tumor*.ti,ab. |
| 5 | Malignanc*.ti,ab. |
| 6 | 1 or 2 or 3 or 4 or 5 |
| 7 | Case Management/ |
| 8 | (case adj2 management).ti,ab. |
| 9 | 7 or 8 |
| 10 | exp Review Literature as Topic/ or Meta-Analysis as Topic/ or Meta-Analysis/ or "systematic review"/ or "Systematic Review".pt. or "Systematic Reviews as Topic"/ |
| 11 | ((systematic adj2 review) or (meta adj2 analysis)).ti,ab. |
| 12 | (((systematic or state-of-the-art or scoping or literature or umbrella) adj (review* or overview* or assessment*)) or "review* of reviews" or meta-analy* or metaanaly* or ((systematic or evidence) adj1 assess*) or "research evidence" or metasynthe* or meta-synthe*).ti,ab. |
| 13 | 10 or 11 or 12 |
| 14 | 6 and 9 and 13 |
